# Age and sex-specific associations between depressive symptoms, body mass index and cognitive functioning among Korean middle-aged and older adults: a cross-sectional analysis

**DOI:** 10.1186/s12877-022-03079-3

**Published:** 2022-05-10

**Authors:** Hyun-E Yeom, Young-Joo Kim

**Affiliations:** 1 College of Nursing, Chungnam National University, Munhwaro 266, Daejeon, 35015 Junggu Korea; 2 Department of Economics, Hongik University, Wausanro 94, Seoul, 04066 Mapogu Korea

**Keywords:** Aging, Body mass index, Cognitive functioning, Depression, Mediation

## Abstract

**Background:**

Although depression and body weight have been noted as important predictors of cognitive health, it remains unclear how age and sex influence the mechanism by which depressive symptoms and body weight are associated with cognitive functioning. This study examined whether and how the relationships between depressive symptoms and cognitive functioning mediated by body mass index (BMI) differ in terms of age and sex.

**Methods:**

A cross-sectional analysis of a large sample of population-based data (*N* = 5,619; mean age 70.73 [± 9.07]), derived from the Korean Longitudinal Study of Aging, was conducted with hierarchical mediated-moderation regressions and a PROCESS macro approach in SPSS. Depressive symptoms were measured through the 10-item Center for Epidemiologic Studies Depression (CES-D) scale, and cognitive functioning was assessed with the Korean Mini-Mental State Examination (K-MMSE).

**Results:**

The results showed that depressive symptoms were significantly associated with cognitive decline directly and indirectly through reduced BMI. The estimated coefficients indicated that a one standard deviation increase in CES-D scale was associated with about 0.9 decrease in K-MMSE score. However, the indirect relationship between depressive symptoms and cognitive function through BMI emerged only in men or individuals older than 70 years.

**Conclusions:**

The findings suggest that a careful assessment of BMI is warranted for early detection and prevention of cognitive decline related to depressive symptoms, particularly among older men.

## Background

An increasing proportion of aged population coupled with an extended life expectancy results in a global trend of aging society [1]. Among the developed countries, some are confronted with a faster growth of the older population. For example, in Korea, adults aged 65 years and older comprise about 16.5% of the population as of 2021 and this proportion is projected to increase to 20.3% in 2025 [2]. The consequent rise in geriatric and healthcare burden is a great challenge to the society. Cognitive decline is one of the health problems accompanied by aging [3]. There have been concerted efforts to identify risk factors of cognitive decline and impairment, apart from aging, for early detection and prevention.

Extensive empirical literature has described the impacts of physical and psychological health on cognitive function, based on the mind–body interaction entangled with hormonal, immunological, and metabolic responses [4–6]. A recent literature review showed that older individuals with depression were more likely to experience cognitive problems related to memory, attention, and learning, all of which are associated with overall executive performance [6]. Two cross-sectional studies of older adults provided strong evidence that depression was a critical factor related to cognitive decline among the aging population [7, 8].

Depression has also been observed as a risk factor for increasing physical vulnerability [9, 10], with regard to dysregulation of biological pathways related to metabolic rates and inactive lifestyles [11]. Specifically, a number of empirical longitudinal studies and meta-analyses have pointed out the link between depressive symptoms and an abnormal body mass index (BMI), mainly in terms of obesity. For example, a meta-analysis of longitudinal studies reported strong evidence that depression contributed to an increased risk of obesity [10, 12]. However, two cross-sectional studies reported that depression could lead to weight loss and being underweight due to appetite loss and malnutrition, eventually associated with cognitive decline among older adults [13, 14].

Further, epidemiological studies have found that the relationship between depression and BMI is heterogeneous, and that it depends on several sociodemographic factors, such as age and sex [15–18]. For example, Lee et al. [17] discovered a U-shaped relationship between depression and BMI particularly in women or the younger age group based on a large cross-sectional study of Korean adults. Liao et al. [18] reported that the link between depression and BMI tended to be stronger with increased age from a large cross-sectional sample of young and older adults in China. However, the prevalence of depression and obesity differ by sex, due to hormone-related physiological processes and other factors [16]. Additionally, several studies have shown distinctive patterns in the relations between depression and weight changes by sex. Li et al.’s [19] cross-sectional study of young and middle-aged adults in the U.S. indicated that the association between depression and obesity existed only in women. Meanwhile, Liao et al. [18] found that the link between depression and being underweight was stronger in men than in women. Therefore, the relationship of depression with BMI based on age and sex differences is inconclusive.

Empirical research has shown a clear link between BMI changes and cognitive functioning. A two-year prospective study indicated that individuals with a lower BMI tended to have a greater risk of developing dementia [20]. However, several studies based on longitudinal data from Europe have warned of an increased risk of cognitive decline caused by a higher BMI during mid to late life [21, 22].

As discussed above, numerous studies suggest BMI is an important indicator that responds to changes in depressive symptoms and is associated with cognitive functioning. However, the role of BMI in the relationship between depressive symptoms and cognitive functioning is uncertain. Specifically, little is known about whether depressive symptoms lead to cognitive decline through BMI changes, and whether the association differs according to age and sex. The heterogeneous relationships between BMI, depressive symptoms, and cognitive decline indicate a need for more sophisticated investigations based on the consideration of their reciprocal interactions using large and population-representative data. Therefore, this study investigated the association between depressive symptoms, BMI, and cognitive functioning, with particular focus on the role of BMI as a potential channel through which depressive symptoms were related to cognitive functioning. The current study also examined whether age- or sex-related differences were present in the association.

### Conceptual model and hypothesized relationship

We constructed a moderated mediation model in which depressive symptoms are associated with cognitive functioning directly and indirectly through BMI (i.e., mediation channel), with heterogeneity by age or sex (i.e., moderation channel).

Our hypothesis for the mediation channel is that depressive symptoms (X) are associated with BMI (M), which is also related to changes in cognitive functioning (Y), postulating that BMI plays a role in mediating the relationship between depressive symptoms and cognitive functioning. The hypothesis for the moderation channel is that the relationship between depressive symptoms and cognitive functioning interacts with BMI contingent on either age or sex (W = moderator).

The model can be succinctly presented as follows:

1 $$\mathrm{Y}={\upbeta }_{1}\mathrm{X}+{\upbeta }_{2}\mathrm{M}+{\uprho }^{^{\prime}}\mathrm{V}+\upvarepsilon ,$$

2 $$\mathrm{M}={\alpha }_{1}\mathrm{X}+{\alpha }_{2}\mathrm{W}+\delta \mathrm{WX}+{\gamma }^{\mathrm{^{\prime}}}\mathrm{V}+\mathrm{u}.$$

The first equation explains how cognitive functioning (Y) is associated with depressive symptoms (X), BMI (M), and covariates (V). The second equation shows that BMI (M) is also associated with depressive symptoms (X), a moderator (W) and the covariates (V), allowing the moderator to interact with depressive symptoms.

The comprehensive channels by which depressive symptoms are associated with cognitive functioning can be decomposed into the direct and indirect channels, and can be obtained through differentiations, as follows:

3 $$\frac{dY}{dX}=\frac{\partial Y}{\partial X}+\frac{\partial Y}{\partial M}\frac{\partial M}{\partial X}={\upbeta }_{1}+{\upbeta }_{2}\left({\alpha }_{1}+\delta W\right).$$

**Fig. 1:**
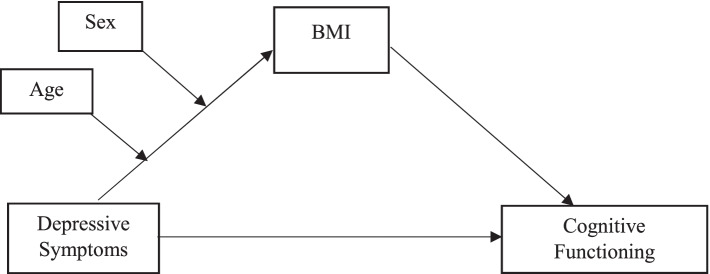
Conceptual model and hypothesized relationship among depressive symptoms, BMI, and cognitive functioning, linked to age and sex

In Eq. (3), the first term ${\upbeta }_{1}$ measures the direct relationship between depressive symptoms (X) and cognitive functioning (Y). The second term ${\upbeta }_{2}\left({\alpha }_{1}+\delta W\right)$ measures the indirect relationship between depressive symptoms and cognitive functioning through BMI, which differs by age or sex $({\alpha }_{1}+\delta W$). The comprehensive channels that describe the links between depressive symptoms, cognitive funtioning, and BMI are illustrated in Fig. 1 below.

## Methods

### Data and procedure

The study sample comprised nationwide, cross-sectional data derived from the recent 7th wave of the Korean Longitudinal Study of Aging (KLoSA), which was collected between September 1 and November 30, 2018. The target population for the current study was adults aged 45 years and older, who had not been medically diagnosed with dementia. The KLoSA data were gathered by region and residence type, proportionate to the local population for quota sampling, and were collected through face-to-face interviews using a computer.

### Measures

Depressive symptoms were measured with the 10-item Center for Epidemiologic Studies Depression (CES-D) scale [23, 24]. The CES-D scale has been widely used to screen depressive symptoms and has been globally validated [25]. The tool consists of 10 items that assess the frequency of experiencing a feeling or thought related to depression. Questions were answered using a 4-point Likert scale (i.e., rarely or never, sometimes, often, always). For this study, each response was recorded as a dichotomous score of 0 for “rarely felt” or “never” and 1 otherwise. A total score of all items was calculated, ranging from 0 to 10, with higher scores indicating greater depressive symptoms.

Cognitive functioning was assessed using the Korean Mini-Mental State Examination (K-MMSE). The MMSE is a globally used measure that assesses cognitive impairment in the clinical and research fields [26, 27]. The K-MMSE is a variation of the MMSE that was adapted and validated for older Koreans [28]. It consists of 11 items categorized into different sections, including orientation (time, place, and person), memorization, attention and calculation, recall, language, and visual construction. The total score, which could range from 0 to 30, was calculated. In general, a score of 17 or lower indicates an increased risk of dementia, while a score of 24 or above indicates normal cognitive functioning.

BMI was calculated using weight and height (kg/m^2^). For covariates, we considered sociodemographic, health-related, and behavioral factors. Sociodemographic characteristics included age, sex, marital status of having a living spouse, and total family income. In terms of health-related characteristics, we included a score for activities of daily living (ADL) and a subjective health status indicator based on a 5-point Likert scale. Behavioral characteristics included indicators of meeting with friends at least once a month and performing regular exercise.

### Statistical analyses

Statistical analyses were performed using SPSS 26.0 (IBM Corp., Armonk, NY, USA) and Stata version 14 (Stata Corporation, College Station, TX, USA). Descriptive statistics were obtained to describe the basic quantitative characteristics of all study variables. Our hypothesized model, including moderation and mediation channels, was tested using the PROCESS macro bootstrapping approach for SPSS [29]. First, the moderation channel of age or sex was tested to detect whether the relationship between depressive symptoms and BMI differed based on either of these. Second, the model incorporated an indirect relationship between depressive symptoms and cognitive functioning through BMI; that is, BMI mediates the relationship between depressive symptoms and cognitive functioning. For an overall assessment of these two features, the model tested whether there were statistically sigificant differences in the indirect relationship between depressive symptoms and cognitive functioning through BMI, according to age and sex.

## Results

### Participants’ sociodemographic characteristics

General characteristics of the participants are shown in Table 1. The average cognitive functioning score of the K-MMSE was 25, with a slightly higher mean for men. For men and women, the average BMI was 23.25 and 23.50, while mean CES-D scores were 2.94 and 3.35, respectively. The mean age was 71 years, which was similar for men and women.

**Table 1:** Descriptive characteristics of the participants by sex

Variables	All		Men		Women	
	*N* = 5,619		*N* = 2,363		*N* = 3,256	
	M ± SD or n (%)		M ± SD or n (%)		M ± SD or n (%)	
Cognitive Function	25.046	± 5.571	25.915	± 5.083	24.415	± 5.820
BMI	23.393	± 2.724	23.250	± 2.502	23.496	± 2.870
CES-D	3.177	± 2.907	2.944	± 2.883	3.346	± 2.912
Age	70.735	± 9.075	70.624	± 8.775	70.815	± 9.287
Family Income^a^	2782	± 2624	7.688	± 0.838	7.479	± 0.937
ADL	0.129	± 0.828	0.110	± 0.765	0.143	± 0.871
Married^b1^	4,197	(74.69)	2,124	(89.89)	2,073	(63.67)
Meet Friends^b2^	4,525	(80.53)	1,883	(79.69)	2,642	(81.14)
Good Health^b3^	1,504	(26.77)	724	(30.64)	780	(23.96)
Regular Exercise^b4^	1,895	(33.72)	897	(37.96)	998	(30.65)

*Abbreviations*: *M* ± *SD* Mean ± standard deviations, *n (%)* Number of observations (percentage)

Cognitive Function is measured with K-MMSE score. BMI is body mass index (kg/m^2^)

CES-D is the CES-D-10 scale for assessment of depressive symptoms

ADL is activities of daily living score

^a^Family Income is annual household income in ₩10,000

^b^Dummy variable indicating group: ^1^ = Currently married with a living spouse; ^2^ = Meet friends at least once a month; ^3^ = have a good health status; ^4^ = Exercise regularly

### Age-related differences in the role of BMI as a mediator

**Fig. 2:**
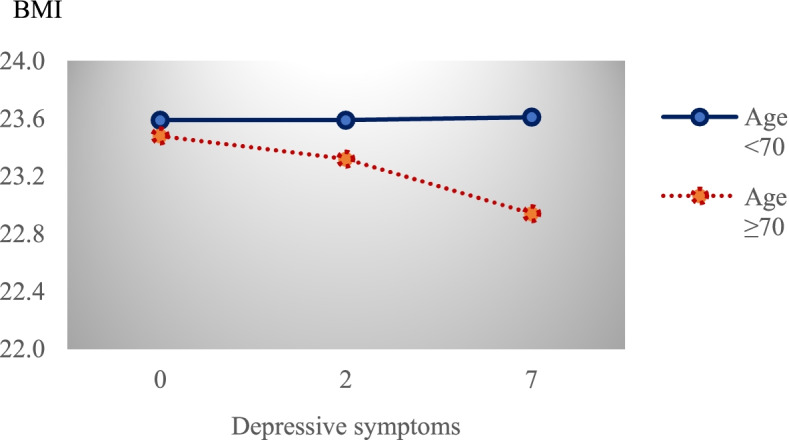
Age-related difference in the relationship between depressive symptoms and BMI

Table 2 presents empirical findings of the path from depressive symptoms to cognitive functioning through BMI when age was a moderator. Overall, the results in Table 2 show that there was a significant interaction between depressive symptoms and age in predicting BMI (*β* = -0.08, *p* = 0.002), confirming age as a moderator in the relationship between depressive symptoms and BMI. Specifically, greater depressive symptoms were associated with a lower BMI, particularly among individuals 70 years or older (see Fig. 2).

**Table 2:** Age-specific direct and indirect relationship between depressive symptoms and cognitive functioning through BMI

Variables	Mediator					Outcome					
	BMI					Cognitive Function					
	$\beta$	SE	*t*		*p*	$\beta$	SE		*t*		*P*
CES-D × Age ≥ 70	-0.080^**^	0.026	-3.149		0.002						
Age ≥ 70	-0.110	0.115	-0.956		0.339						
CES-D	0.004	0.019	0.192		0.848	-0.323^**^	0.023		-14.352		0.000
BMI						0.139^**^	0.022		6.204		0.000
Covariates											
Sex^a1^	-0.319^**^	0.077	-4.134		0.000	0.614^**^	0.129		4.758		0.000
Family Income^b^	0.125^**^	0.046	2.720		0.007	0.958^**^	0.072		13.253		0.000
ADL	-0.139^**^	0.045	-3.099		0.002	-2.084^**^	0.075		-27.884		0.000
Married^a2^	0.094	0.092	1.015		0.310	1.431^**^	0.153		9.328		0.000
Meet friends^a3^	-0.082	0.095	-0.866		0.387	1.777^**^	0.160		11.134		0.000
Good health^a4^	-0.198^**^	0.086	-2.291		0.022	1.317^**^	0.142		9.254		0.000
Regular Exercise^a5^	0.151^*^	0.077	1.948		0.052	1.144^**^	0.130		8.804		0.000
	R^2^ = 0.020, F (10, 5608) = 11.547, *p* < .000					R^2^ = 0.340, F (9, 5609) = 320.456, *p* < 0.000					
	Indirect relationship between Depressive Symptoms and Cognitive Function through BMI										
Age	Estimate			SE				95% LLCI		95% ULCI	
Age < 70	0.001			0.003				-0.005		0.006	
Age ≥ 70	-0.011^**^			0.003				-0.018		-0.005	
	The Difference in Indirect relationship across Two Age Groups										
	Estimated difference			SE				95% LLCI		95% ULCI	
(Age ≥ 70)—(Age < 70)	-0.0111^**^			0.0042				-0.0203		-0.0037	

*Abbreviations*: *SE* Standard errors, *t* is *t*-statistics and *p* is the corresponding *p*-value

^a^Dummy variable indicating group: ^1^ = Men; ^2^ = Currently married with a living spouse; ^3^ = Meet friends at least once a month; ^4^ = have a good health status; ^5^ = Exercise regularly

^b^Logarithm of family income is used in the estimation model

Standard errors are bootstrapped using 5,000 bootstrap replications

^**^ *p* < 0.05; ^*^ *p* < 0.10

Depressive symptoms were a significant predictor of cognitive functioning (*β* = -0.323, *p* = 0.000), as shown in Table 2. Given the standard deviation of CES-D scale in Table 1 (σ = 2.907), the estimated coefficients in Table 2 indicate that a one standard deviation increase in CES-D scale was associated with 0.94 decrease in K-MMSE score. The indirect relationship between depressive symptoms and cognitive functioning through BMI differed by age. For adults aged 70 and older, the relationship was significant (indirect relationship = -0.011, 95% confidence interval [CI] = -0.018, -0.005). Contrastingly, this indirect link was not found in adults younger than 70 years.

The estimated difference in the indirect relationship across the two age groups was significant (Estimated difference = -0.0111, 95% CI = -0.0203, -0.0037). The results indicate that the indirect relationship between depressive symptoms and cognitive functioning through BMI emerged only among individuals aged 70 years and older.

### Sex-related differences in the role of BMI as a mediator

**Fig. 3:**
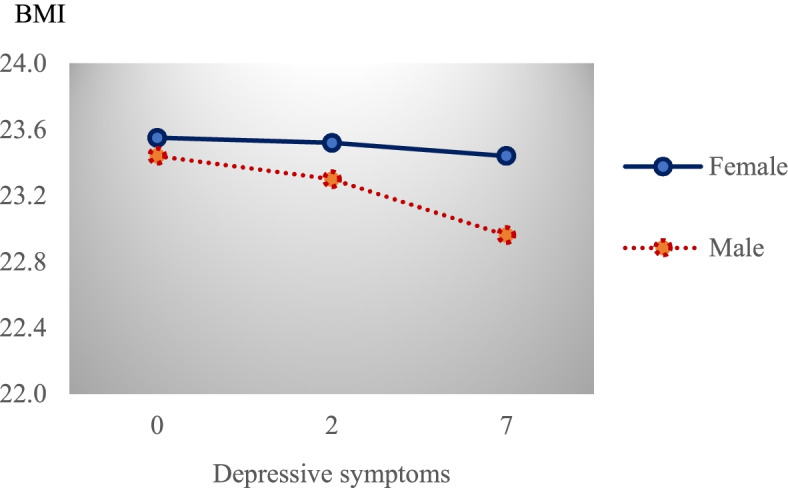
Sex-related difference in the relationship between depressive symptoms and BMI

Table 3 presents the estimation results when sex was included as a moderator. A significant interaction between depressive symptoms and sex was detected in predicting BMI (*β* = -0.053, *p* = 0.035), which was also noted in Fig. 3.

**Table 3:** Sex-specific direct and indirect relationship between depressive symptoms and cognitive functioning through BMI

Variables	Mediator					Outcome					
	BMI					Cognitive Functioning					
	$\beta$	SE	*t*		*p*	$\beta$	SE		*t*		*p*
CES-D × Sex^a1^	-0.053^**^	0.025	-2.105		0.035						
Sex^a1^	-0.115	0.109	-1.051		0.294						
CES-D	-0.016	0.017	-0.916		0.360	-0.297^**^	0.022		-13.833		0.000
BMI						0.076^**^	0.021		3.549		0.000
Covariates											
Age	-0.037^**^	0.005	-7.584		0.000	-0.190^**^	0.008		-24.637		0.000
Family income^b^	0.060	0.046	1.292		0.197	0.293^**^	0.074		3.963		0.000
ADL	-0.115^**^	0.045	-2.560		0.011	-1.800^**^	0.072		-24.993		0.000
Married^a2^	0.006	0.094	0.065		0.948	0.908^**^	0.144		6.327		0.000
Meet friends^a3^	-0.111	0.095	-1.173		0.241	1.632^**^	0.152		10.745		0.000
Good health^a4^	-0.280^**^	0.086	-3.251		0.001	0.682^**^	0.138		4.946		0.000
Regular exercise^a5^	0.147^*^	0.077	1.902		0.057	1.196^**^	0.124		9.681		0.000
	R^2^ = 0.026, F (10, 5608) = 14.830, *p* < .000					R^2^ = 0.402, F (9, 5609) = 418.371, *p* < 0.000					
	Indirect relationship between Depressive Symptoms and Cognitive Function through BMI										
Sex	Estimate			SE				95% LLCI		95% ULCI	
Women	-0.001			0.002				-0.005		0.002	
Men	-0.005^**^			0.002				-0.010		-0.002	
	The Difference in Indirect relationship across Men and Women										
	Estimated difference			SE				95% LLCI		95% ULCI	
Men-Women	-0.0040^**^			0.0024				-0.0094		-0.0001	

*Abbreviations*: *SE* Standard errors, *t* is *t*-statistics and *p* is the corresponding *p*-value

^a^Dummy variable indicating group: ^1^ = Men; ^2^ = Currently married with a living spouse; ^3^ = Meet friends at least once a month; ^4^ = have a good health status; ^5^ = Exercise regularly

^b^Logarithm of family income is used in the estimation model

Standard errors are bootstrapped using 5,000 bootstrap replications

^**^ *p* < 0.05; ^*^ *p* < 0.10

The results in Table 3 also show how depressive symptoms and BMI were related to cognitive functioning. The direct link between depressive symptoms and cognitive functioning was significant (*β* = -0.297, *p* = 0.000) after adjusting for the covariates and BMI. Given the standard deviation of CES-D scale in Table 1 (σ = 2.907), the estimates in Table 3 imply that a one standard deviation increase in depression scale was associated with 0.86 decrease in K-MMSE score.

The lower panel of Table 3 shows that the indirect relations between depressive symptoms and cognitive function through BMI differed by sex. For men, depressive symptoms were significantly associated with cognitive functioning (indirect relationship =-0.005, 95% CI = -0.010, -0.002), but the estimated relationship for women was insignificant. The sex-related difference in the estimated relationship was significant (estimated difference = -0.004, 95% CI = -0.0094, -0.0001).

## Discussion

This study investigated the sophisticated associations between depressive symptoms, BMI, and cognitive functioning, with a focus on the role of BMI in mediating the relationship between depressive symptoms and cognitive functioning, as well as age- and sex-related differences. Several important findings are comparable to those of the existing empirical literature on the relationship between depressive symptoms, BMI, and cognitive functioning.

One of our key findings is that greater depressive symptoms were related to a lower BMI. This result substantiates the empirical findings concerning the association between depression and being underweight later in life as observed in previous literature [13, 14, 18]. In particular, our findings are consistent with those of previous studies from Asian countries, including China and Korea, which found an inverse relationship – a higher risk of depression in association with being underweight [15, 30]. This association is noteworthy, because the direction of the relationship is contradictory to the pattern observed in prior research, which has often identified obesity as a problem related to depressive symptoms [10, 16]. For example, a meta-analysis of longitudinal studies from Western countries confirmed the reciprocal relationship between depression and obesity [10].

What is also noteworthy from our findings is that the relationship between depressive symptoms and BMI was moderated by sex. The link between the two was much stronger among men. Furthermore, an age-related difference in this relationship was also found. For people older than 70 years, BMI tended to decrease more steeply, along with an increase in depressive symptoms. These findings are consistent with those of a recent study conducted using a large Chinese sample [18]. In this Chinese study, the relationship between depressive symptoms and being underweight was more evident in men than women, and this tendency increased with age. The sex-specific relationship between depression and BMI was also found among a Western population [19]. In general, women were more likely to be depressed and obese than men, with some exceptions [30]. The different patterns of association between depressive symptoms and BMI across regions indicate a need for further investigation, with careful consideration being given to various sociocultural environments, to obtain a clear and comprehensive picture of this relationship.

The other key finding of this study is that BMI played a role in mediating the relationship between depressive symptoms and cognitive functioning only in men or people older than 70 years. Although depressive symptoms were associated with deteriorating cognitive functioning in all age groups and sexes, the indirect relationship between depressive symptoms and cognitive functioning through BMI was found only in men and people older than 70 years. For women or people younger than 70 years, an increase of depressive symptoms was a primary contributor to declining cognitive functioning, regardless of the influence of BMI. These findings provide a practical implication that an assessment of BMI could be applied not only as a fundamental step to screen depressive symptoms, but also as a useful means for the early detection of cognitive decline related to depression, particularly in men and adults older than 70 years.

The results of this study—that the role of BMI varies by age and sex—fill a gap in the literature that has previously reported inconsistent patterns regarding the relationship between BMI and cognitive functioning. Cova et al.’s [31] study found that higher BMI had a protective effect on the risk of cognitive deficits, while Singh-Manoux et al.’s [21] study indicated that a higher BMI during midlife was a risk factor for late-life dementia. Some studies that had considered age in BMI’s influence on cognitive functioning also presented contrasting results. For instance, a longitudinal study showed that a higher BMI during late life had a protective effect against dementia [32]. Contrastingly, another study found that a higher BMI during midlife and a decrease in BMI during later life increased the risk of dementia [22]. These differing results imply that life-course observations from middle-aged to older adults are necessary to reach a confirmed conclusion. Therefore, the findings of the current study highlight the importance of a careful assessment of BMI and the need to maintain adequate BMI ranges. Specifically, we propose BMI monitoring strategies tailored to age for early detection and prevention of cognitive decline in an aging population with depressive symptoms.

This study contributes to the literature by providing evidence on a refined mechanism involving the specific role of BMI in the relationship between depressive symptoms and cognitive functioning. Nevertheless, this study has some limitations. First, considering that the current study applied simultaneous equation estimation methods to cross-sectional data, our results do not confirm the causal influence of depression on BMI and cognitive functioning. Second, this study uses MMSE scores as a single measure to assess cognitive functioning. Although the MMSE is a popularly used and validated tool to assess overall cognitive function, specifically to screen cognitive impairment and dementia, it is limited to confirming the overall performance of cognitive functioning. Depressive symptoms were also measured with a single instrument of the CES-D scale. The CES-D is a basic screening tool for initial detection of depression risk based on self-reported responses, and therefore there is a lack of clinical confirmation of depression. Third, depressive symptoms and BMI are both known to be associated with multifaceted physiological and socio-behavioral characteristics, such as hormonal and metabolic responses, physical activities, and social interactions [33, 34]. About the complex relationship between depressive symptoms and BMI, our findings are convincing because we also considered socio-behavioral factors—such as exercise and social interactions—that could be entangled with the link between depressive symptoms and BMI. However, only a few biological indicators are included in the current study. Therefore, future research that incorporates physiological, behavioral, and sociodemographic factors is warranted to illuminate the mechanism that explains the link between depression and BMI in an aging population. The last limiting point of this study is that our social interaction and physical activity variables are not measured with validated tools. One may raise the question of whether meeting a friend at least once a month or doing regular exercise are informative indicators of social and behavioral rhythms, which are emphasized in recent literature. As one way of reflecting this point, future work may need to apply validated objective assessment tools that have been discussed in Cleland et al. [35], Carta et al. [36], and Carta et al. [37].

## Conclusions

Depression is a critical risk factor related to the deterioration of cognitive functioning in an aging population. The current study provides a new insight to this link by showing that BMI mediated the relationship between depressive symptoms and cognitive functioning, in particular for men and people older than 70 years. From a policy perspective, a thorough assessment and close monitoring of BMI is useful for the most susceptible group of aging population to capture signs of cognitive decline at an early stage.

## Data Availability

The KLoSA datasets analyzed for the current study are de-identified and are publicly available in the following repository. https://survey.keis.or.kr/eng/klosa/klosa01.jsp.
